# Cortical Activation to Action Perception is Associated with Action Production Abilities in Young Infants

**DOI:** 10.1093/cercor/bht207

**Published:** 2013-08-23

**Authors:** Sarah Lloyd-Fox, Rachel Wu, John E. Richards, Clare E. Elwell, Mark H. Johnson

**Affiliations:** 1 Centre for Brain and Cognitive Development, Birkbeck, University of London, London, Greater London WC1E 7HU, UK; 2 Department of Brain and Cognitive Sciences, University of Rochester, Rochester, NY 14627, USA,; 3 Department of Psychology, University of South Carolina, Columbia, SC 29208, USA; 4 Department of Medical Physics and Bioengineering, University College London, London WC1E 6BT, UK

**Keywords:** action, fNIRS, infancy, IPL, pSTS

## Abstract

The extent to which perception and action share common neural processes is much debated in cognitive neuroscience. Taking a developmental approach to this issue allows us to assess whether perceptual processing develops in close association with the emergence of related action skills within the same individual. The current study used functional near-infrared spectroscopy (fNIRS) to investigate the perception of human action in 4- to 6-month-old human infants. In addition, the infants' manual dexterity was assessed using the fine motor component of The Mullen Scales of Early Learning and an in-house developed Manual Dexterity task. Results show that the degree of cortical activation, within the posterior superior temporal sulcus—temporoparietal junction (pSTS-TPJ) region, to the perception of manual actions in individual infants correlates with their own level of fine motor skills. This association was not fully explained by either measures of global attention (i.e., looking time) or general developmental stage. This striking concordance between the emergence of motor skills and related perceptual processing within individuals is consistent with experience-related cortical specialization in the developing brain.

## Introduction

Our hands are a gateway to both our physical and our social world. Not only do we use our hands to touch and explore our surroundings, we also use them to gesture and convey social information. Understanding one's own and other's actions may be intrinsically linked. Debate on the interplay between perception and action has a longstanding heritage (Descartes 1664; Mach 1922). More recently, Prinz (1997) provided a theoretical framework for the understanding of this functional relationship suggesting that a common representational domain exists between the perception of an action and the production of the same action—a hypothesis that has been supported by a number of studies (Brass et al. 2001; Hamilton et al. 2004). Despite the extensive study of perception-action relations in the brain, a number of critical questions remain. For example, do we require experience of moving our own bodies others moving their body in the same way, or is visual experience of the observation of other peoples' movements sufficient to develop an understanding of human motion and actions in others? This fundamental question can be better understood by turning to the study of infants. Given that perception and action both develop during infancy, the aim of this study was to measure brain activation resulting from the perception of actions during a period when infants are in rapid transition in terms of motor development.

Our fine motor skills are developing from a very early age. From as early as 14 weeks after conception, we are already beginning to perform reliable grasping actions with our hands in utero. Research with preterm neonates, born 2 months premature, has shown that they are able to distinguish between different sized objects in a grasping habituation task—measured by an increased holding time to a novel object following habituation to a familiar object—illustrating an early sensitivity to manual touch (Lejeune et al. 2010). Furthermore, within the first 6 months—in hearing infants with hearing-impaired parents who communicate with sign language—the development of manual gestures is correlated with the observation of hand movements in others (Petitto et al. 2001). Moreover, a study with 6- and 12-month olds suggests that infants are only able to display anticipatory eye movements during a goal-directed action when they are able to perform such an action themselves (Falck-Ytter et al. 2006). In accordance with these findings, grasping performance in 6-month olds during an action production task has been linked with visual performance on a preferential looking discrimination task of object-related human grasping actions (Daum et al. 2011). Finally, performance on a visual discrimination task of goal-directed actions is improved when infants with very little grasping experience (3 months of age) are given self-experience of grasping using Velcro “sticky” mittens (Sommerville et al. 2005).

Human action perception in adults has been associated with cortical processing over an extensive portion of the cortex, including the ventral premotor cortex (including inferior frontal gyrus) and regions of the parietal and temporal cortex comprising the superior temporal sulcus (STS), inferior parietal lobule (IPL), and temporoparietal junction (TPJ). These cortical regions have been identified during a wide range of human action paradigms in adults, such as biological motion perception, goal-directed actions, and intention attribution (Allison et al. 2000; Pelphrey et al. 2005; Van Overwalle and Baetens 2009). Anatomically, visual input regarding human biological movement is processed in the STS and then passed to the IPL, TPJ, premotor, and frontal areas. Single-cell recording studies with macaques have revealed neurons that are responsive to actions of others and/or themselves in the STS (Perrett et al. 1989), ventral premotor cortex (Di Pellegrino et al. 1992), and IPL (Fogassi et al. 2001; Gallese et al. 2002; Rizzolatti and Craighero 2004). These studies have shown that neurons can have strictly visual responses (i.e., they respond to the perception of specific actions in others; (Perrett et al. 1989)), strictly congruent responses (i.e., they respond to observations of a specific action that the individual can also do themselves), or broadly congruent responses (i.e., they respond to the observation of a diverse array of actions and not only ones that the individual can perform themselves) (Liew and Aziz-Zadeh 2013). Some of these neurons in the premotor cortex and IPL have been termed “mirror neurons” for their ability to respond to both an action that the individual performs and mirrored observations of that same action in others (Rizzolatti and Craighero 2004).

The processing of a perceived action has been attributed to several hypothetical systems, including the action observation network (AON) and the mentalizing system. Many researchers have suggested that particular neural systems become active depending on the specific context or task (Keysers and Gazzola 2007; Van Overwalle and Baetens 2009; Liew et al. 2011). The AON (which includes the putative human mirror neuron system) is thought to have general action observation response parameters, encompassing a broad network of brain regions (Cross et al. 2008). The AON allows us to recognize the goal of a perceived action of another, by matching this with representations of ones own actions (Van Overwalle 2009). Therefore, it is commonly assumed to be limited to familiar and frequently executed actions (Calvo-Merino et al. 2005; Cross et al. 2006) and responds more to human than nonhuman action (Pelphrey et al. 2003). In contrast, the mentalizing or Theory of Mind (ToM) system (i.e., Frith and Frith 2006) is generally thought to be involved in the intentionality, communicative content, and/or social relevance of the action. It is thought that the engagement of the mentalizing system is dependent on the type of action observed and likely comes online in the presence of goal-directed or communicative cues that require reflective metacognition (Aziz-Zadeh et al. 2012). It has recently been suggested that biological tuning to human actions, relative to nonbiological movements, arises through an associative learning mechanism because the human actions have been observed more frequently while executing corresponding actions (Press 2011). If specialization within the AON to actions relies on associative learning between the perception and production of similar actions, then one might expect a greater degree of activation when perceiving actions that have been physically experienced, such as to dance in expert dancers or to walking in infants who are able to walk.

Despite extensive developmental behavioral studies of action perception and production, this work does not allow direct inferences about common substrates in the brain. To measure this in infancy, it is crucial that we investigate action production, perception, and associated brain correlates within the same individuals. Several recent studies in infants have reported activity in the posterior temporal region of the cortex in relation to the perception of human actions, including communicative eye gaze shifts, mouth movements, and actions of the hand (Grossmann et al. 2008; Lloyd-Fox et al. 2009, 2011; Ichikawa et al. 2010). However, it is not known whether the recruitment of this region for action perception is affected by infants' own experiences. The posterior temporal brain region identified in these infant studies lies approximately over the pSTS-TPJ region (hereafter, we use the term “pSTS-TPJ region” to refer to regions of the posterior superior temporal gyrus and sulcus extending through the TPJ to the IPL). These brain areas have been consistently associated with research on human actions in adults (Van Overwalle and Baetens 2009). Within this region, while the pSTS and IPL are often linked to the AON and therefore the processing of biological motion, action observation, and intentionality (Allison et al. 2000; Pelphrey et al. 2005), the TPJ is usually linked with the mentalizing system and the transient reasoning of goals and intentions (Van Overwalle 2009). However, as acknowledged in a meta-analysis of studies that investigated these action-associated systems (Van Overwalle and Baetens 2009), it is often difficult to dissociate the TPJ and pSTS in adult fMRI work, and there is no clear consensus on their anatomical definitions.

Given that the pSTS-TPJ region is involved in the visual interpretation of actions and socially complex scenes in adults, and is known to change with new experiences of actions (Cross et al. 2009), we aimed to investigate this association during early infant development. As behavioral studies have shown that fine motor experience in infants is associated with an ability to interpret perceived actions in others (Sommerville et al. 2005; Falck-Ytter et al. 2006; Gredebäck and Melinder 2010; Daum et al. 2011), we aimed to investigate whether in those infants with better fine motor skills, the pSTS-TPJ region may be activated to a greater degree when viewing others perform manual actions. In other words, we hypothesize that the development of infants' own fine motor actions is associated with a bias to look for, and therefore process similar actions in others.

Investigating localized cortical activation within individual infants is challenging and has been rarely undertaken due to constraints in available methodology. Functional near-infrared spectroscopy (fNIRS) provides an elegant solution to bridge this methodological gap, and is emerging as an important new technology for investigating developmental cognitive neuroscience (for a review of infant work, see Lloyd-Fox et al. 2010). Similar to fMRI, fNIRS measures hemodynamic responses to neuronal activation. Although fMRI has superior spatial resolution compared with fNIRS, research from adults has shown a high degree of correlation between simultaneous recordings of hemodynamic responses with fNIRS and fMRI (Steinbrink et al. 2006).

**Figure 1.**
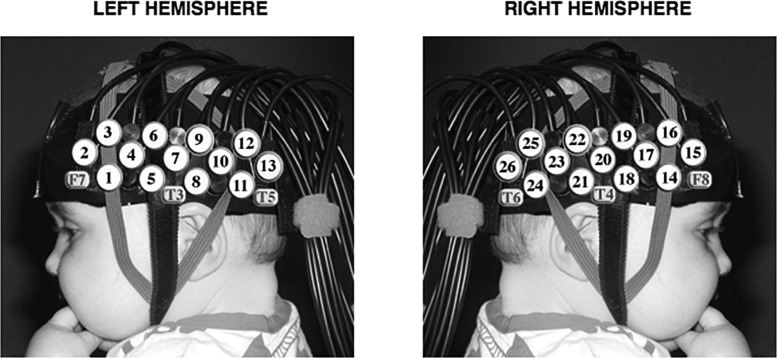
An infant wearing the fNIRS headgear with channel locations and the locations of the 10–20 coordinates on an average 4- to 6-month-old head displayed.

We used fNIRS to investigate neural substrates within individual infants and explore emerging relationships between early developing patterns of brain activation in response to the perception of human manual actions in others, and the infants' own fine motor development (Fig. 1). Infancy is an ideal stage in the lifespan to investigate these issues, as motor ability is rapidly improving. Prior to the onset of crawling, functional and communicative manual gestures are one of the primary strategies that infants can employ to interact with their environment. The investigation of infants from 4 to 6 months—an age when they begin to plan and coordinate their actions and use their hands to willfully and independently grasp objects—is an ideal age for assessing this relationship (Von Hofsten and Lindhagen 1979; Woodward 1998). Within this age group, we should find infants with a considerable range of fine motor skills.

We first used NIRS to measure cortical responses resulting from viewing human adult manual actions to investigate whether parts of infants' posterior temporal cortices are sensitive to such actions (Lloyd-Fox et al. 2011). Following this, we assessed the same infants' fine motor skills using a manual dexterity task and/or the motor components of the Mullen Scales of Early Learning (Mullen 1995) to investigate the association between perceptual and motor development at 4–6 months of age. We propose that perceptual processing develops in close association with the emergence of related action skills within the same individual. Therefore, we predicted that activation to the perception of manual actions (but not to a control condition of eye gaze shifts) in the pSTS-TPJ region of the cortex would be correlated with action production by 6 months of age.

## Experimental Procedures

### Participants

Twenty-four healthy 4- to 6-month-old infants (10 females; mean age, 154.8 days; range, 134–174 days) participated in this study. A further 12 infants participated but were excluded from the study, as they did not watch a sufficient number of trials during the perceptual human action fNIRS task (*N* = 8), did not complete the behavioral measures of motor development (*N* = 2), or too many channels (>40%) were rejected by the artifact detection algorithms and analyses (*N* = 2). This relatively high level of attrition was due in part because of the length of the study session—3 tasks—which was difficult for young infants to complete, and because some infants became fussy during the fNIRS (caused in some cases by the addition of an initial eye-tracking calibration period before the onset of the study). Despite this, the attrition rate is still within the typical range for infant fNIRS studies (Lloyd-Fox et al. 2010). All parents gave informed consent before the study, and the ethics committee at Birkbeck, University of London approved the study design.

### fNIRS Paradigm

#### Stimuli

The experimental design comprised 2 experimental conditions and 1 baseline condition. The experimental stimuli consisted of full color approximately life-size video clips of female actors (head and shoulders in display) in 2 conditions: 1) manual action condition—the actors moved their hand by closing their palm to form a fist and opening again and by retracting and flexing their fingers from 1 to 5; and 2) eye gaze shift condition—the actors moved their eyes left, right, opened, or closed (Lloyd-Fox et al. 2011). The baseline condition consisted of a sequence of full-color still images of different types of transport (i.e., cars, helicopters) presented randomly for a pseudorandom duration (1–3 s) (Lloyd-Fox et al. 2009). The baseline condition provided a reference response from which to compare the activated period during the experimental conditions. The overall surface area of the stimuli was equivalent and extended a maximum visual angle of 16.1°. To maintain attention, occasional alerting sounds and a “high-interest” video (Peek-a-boo) were used to draw the infant's attention back to the screen.

#### Procedure

Infants wore custom-built NIRS headgear consisting of 2 source-detector arrays (see Fig. 1) containing a total of 26 channels (source: detector separations; 20 mm) and were tested with the UCL topography system (NTS2; Everdell et al. 2005). This system used 2 continuous wavelengths of source light at 770 and 850 nm. Based on an understanding of light transport and given that the cortex is ∼0.5 cm from the skin surface in this age group (Salamon et al. 1990), the channel separations used in the current study were predicted to penetrate up to a depth of ∼2 cm from the skin surface, potentially allowing measurement of both the gyri and parts of the sulci near to the surface of the cortex. Before the infants began the study, measurements of their head circumference, and distance between glabella, ears, and inion were taken, and the location of the channels and arrays relative to these anatomical landmarks were recorded. The distance from the midpoint of the headband over the forehead (the glabella) to the midpoint of the temporal arrays (channel 9; left hemisphere and 28; right hemisphere) is fixed at 11 cm and is aligned approximately with T3 and T4 of the 10–20 system on an average 5-month-old infant head. Measurements from this group of 4- to 6-month-old infants showed that the average head circumference was 42.94 cm, and the average distance from the glabella to T3/T4 was 11.64 cm [standard deviation (SD) 0.47].

The infants sat on their parent's lap in front of a Tobii 1750 eye-tracker (www.tobii.com). The stimuli were presented on the 17-in monitor attached to the eye-tracking unit using Tobii's ClearView AVI presentation software with sounds played through stereo external speakers. Prior to starting the experiment, the experimenter used the standard 5-point calibration on the infants' looks (von Hofsten et al. 2005; Wu and Kirkham 2010).

The trials alternated one after the other, beginning with a 10-s baseline trial followed by a 10-s experimental trial. The 2 experimental conditions were presented pseudorandomly to prevent anticipatory effects, and to ensure the infant was presented with an equal number of trials per condition after every 10 trials. During the stimulus presentation, the trials were spliced with attention enhancers (stationary kaleidoscope images and a bell sound) to maintain infant attention.

#### Data Processing, Rejection, and Analysis

Within each optical array, light reaching the detectors travels from the sources through the skin, skull, and underlying brain tissue. The NIRS system measures the absorption of this light, from which the changes in oxy-hemoglobin (HbO_2_) and deoxy-hemoglobin (HHb) concentration (µMol) are calculated and used as hemodynamic indicators of neural activity (Obrig and Villringer 2003). The method of data processing, rejection, and analysis of the optical data follows an established procedure used in previous research (Blasi et al. 2007; Lloyd-Fox et al. 2009, 2011). Either a significant increase in HbO_2_ concentration, or a significant decrease in HHb, is commonly accepted as an indicator of cortical activation in infant work (Lloyd-Fox et al. 2010). If HbO_2_ and HHb were to either increase or decrease significantly in unison, the signal was considered unreliable and not reported in the analyses. Though we could detect significant decreases in the HHb signal, these did not survive correction for multiple comparisons. A time window was selected between 4- and 12-s postexperimental stimulus onset. This period of time was selected to include the range of maximum concentration changes observed across infants for HbO_2_ and HHb. Statistical comparisons of the response to experimental baseline trials were made using the valid data for each channel within each individual.

Inclusion criteria required each channel to contain valid data in a minimum of 3 trials per condition. For a trial to be considered valid, the infant had to be looking at the screen for a minimum of 50% of the experimental trial. This was calculated both from the eye-tracking data (see Supplementary Table 2) and from coding of the videos offline by an experimenter unfamiliar with the study aims. It should be noted that while the group average total look per valid trial was 7.2 s (SD 1.1), for the eye-tracking data, it was 8.8 s (SD 0.84) for the video-coded data. This discrepancy is most likely due to short periods of unstable tracking and fixation detection/rejection algorithms not applied to video hand coding. Though the discrepancy is relatively small, validity of data inclusion was assessed using the video-coded data given its more reliable measure of total looking time. Therefore, validity of data inclusion was assessed using the video-coded data. Across the group of infants, the average number of valid trials per condition was 5.24 (SD 1.56) for the manual action condition and 4.94 (SD 1.75) for the eye gaze shift condition.

The total looking times to the areas of interest (AOIs) and screen were recorded with the eye-tracker and compiled by Tobii's ClearView analysis software (e.g., Wu and Kirkham 2010). Fixations that were shorter than 100 ms were excluded from the final analyses. Proportional looking time to the AOIs in each trial type for every infant was calculated by dividing the total looking time to that location (face or hand) by the total looking time to every location on the screen. Proportional looking measures are a better proxy for looking patterns in infants, as total looking time could be affected by additional factors (such as calibration errors or movement in response to particular stimuli) while proportional looking is more likely to be consistent across trials and infants. The measures of proportional looking were compared with the hemodynamic response in the pSTS-TPJ region of interest (ROI) for each condition using a partial correlation analysis, to control for total looking time per condition.

#### Defining the Region of Interest Using Infant MRI

**Figure 2.**
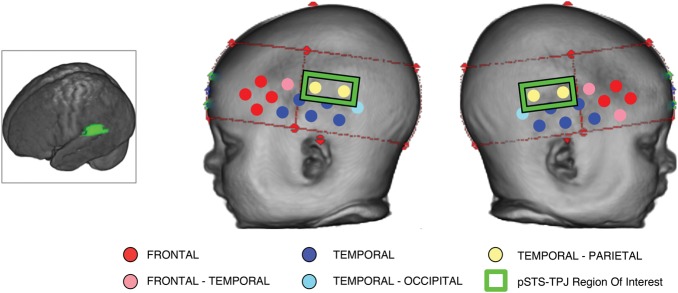
Co-registration of the location of NIRS channels on anatomy using MRI volumes. The main panel shows a reconstructed infant head (from their own MRI) with the position of the NIRS channels overlaid onto the surface. The color-coded channels illustrate the identity of the underlying anatomy beneath each channel across a group of 53 4- to 6-month-old infants (who participated in a NIRS and MRI study; see Blasi et al. 2011; Lloyd-Fox et al. 2011; Lloyd-Fox et al. 2013). The channels contained within the green boxes are positioned over the pSTS-TPJ region (as highlighted in the infant brain image on the left) and form the ROIs for this study.

To conduct ROI analyses on the pSTS-TPJ region (posterior STS/STG and IPL bordering the TPJ), we used a dataset of 53 4- to 6-month-old infants who each had structural MRIs (the MRI images came from the Centre for Neuroimaging Sciences, Kings College London (Blasi et al. 2011)) and recordings of the location of the 26 NIRS channels on their head (from a study at the Centre for Brain and Cognitive Devleopment, Birkbeck College (Lloyd-Fox et al. 2012, 2013)). These infants were from a different dataset to the current study. Co-registration—of the locations of the NIRS channels on the scalp surface with the closest underlying cortical areas—was undertaken using individual infants' MRI volumes. The methods for this are explained in detail elsewhere (Phillips et al. submitted; Richards unpublished manuscript). In brief, for each infant the scalp locations of the NIRS channels were co-registered with reconstructions of the head (obtained from *T*_2_-weighted MRIs (0.7 × 0.7 × 6)) by using fiducials, head measurements, photographs of the NIRS headgear on the head (from front, and each side), and the identification of anatomical landmarks in the reconstructed MRI image. The scalp locations of the NIRS channels were then projected inward to the cortex and stereotaxic atlases were used to identify the lobar and macroanatomical area of the projected location. Figure 2 shows a reconstructed infant head with the position of the NIRS channels overlaid onto the surface. The color-coded channels in the figure illustrate the identity of the underlying cortical area beneath each channel across the group of 53 infants; frontal, frontal-temporal, temporal, temporal-occipital, and temporal-parietal. In those channels which sat over an area of the brain on the border between 2 regions, there is a double label—i.e., frontal-temporal—as in some of the infants, this channel was over their frontal lobe while in others it lay above their temporal lobe. To calculate which channels were over the pSTS-TPJ region, the distance from the TPJ (defined as the posterior superior temporal and middle temporal gyrus bordering with the inferior supramarginal and angular gyrus, see Fig. 2) was calculated for each of the 26 channels. Channels 9, 12, 22, and 25 (within the green boxes on Fig. 2) are positioned on average within 2.92 mm (SD 3.39 mm) of the junction of the TPJ with a median distance of 1.41 mm. Therefore, these channels were used as our pSTS-TPJ ROIs over the left and right hemisphere for the data analyses of the current dataset.

### Behavioral Paradigms: Manual Dexterity Task and Mullen Scale of Early Learning

#### Procedure

The infants sat on their parent's lap at a table while an experimenter sat on the opposite side. The experimenter spoke to the infants to attract their eye contact, then placed an object on the table within reach of the infant and aligned with their midline. The infant was encouraged verbally to engage with the object. The experimenter waited until the infant had grasped the object, resetting it in position if it was knocked out of reach. If the session with each object lasted more than 1 min, the experimenter removed the object and moved on to the next item. There were 4 items in total: a doll's cup, a block, a doll's brush, and a Lego duplo piece (toys were between 2.5 and 4 cm wide and could easily fit into infants' hands). The task was coded offline by an independent coder, who was unfamiliar with the experimental paradigm and hypotheses. To be considered successful, a grasp must lift the object off of the table. The grasp type was assigned a rank from 1 to 6 as follows—(6) hand pincer; (5) whole-hand grasp/2-hand grasp (adult-like); (4) partial finger grasp (i.e., some fingers placed awkwardly); (3) 2-hand grasp inefficient (balanced on fingers of both/ fragile grip); (2) object to torso grasp (uses hand against torso to scoop up object); (1) failure to lift object despite attempts. The grasp was only considered valid if the infant was looking at the object when they attempted to grasp it with their hand(s). Furthermore, the data from each infant was only considered valid if at least 3 of the 4 objects were interacted with, and an average rank could be calculated—if the infant became fussy and the study was ended prematurely then the data were excluded. A second coder then coded the video recordings, and any discrepancies were examined and agreed upon.

Finally, the infants undertook a developmental assessment using the Mullen Scale of Early Learning (Mullen 1995). This assessment included fine motor, gross motor, visual reception, receptive language, and expressive language scales. This was chosen to provide a standardized overall measure of development, and an additional standard measure of fine motor development in addition to the manual dexterity scores.

## Results

In an initial channel-by-channel analysis of the fNIRS data, *t*-tests compared the grand averaged hemodynamic peak changes in HbO_2_ and HHb (during the time window of activation described in the methods) evoked by the manual action and eye gaze shift conditions relative to baseline. To resolve statistical problems of multiple comparisons for the group analyses, we applied the false discovery rate correction (Benjamini and Hochberg 1995). In summary, the manual action condition revealed significant effects in 8 channels (channels: 1: *t* = 3.63, *P* = 0.001; 5: *t* = 2.85, *P* = 0.009; 9: *t* = 3.58, *P* = 0.002; 13: *t* = 3.34, *P* = 0.003; 14: t = 4.81, *P* < 0.001; 15: *t* = 4.93, *P* < 0.001; 28: *t* = 6.67, *P* < 0.001, and 32: *t* = 5.14, *P* < 0.001) located over anterior and posterior portions of the arrays (see Supplementary Materials). A similar pattern of activation was found in response to the eye gaze shift condition (see Supplementary Materials), which revealed significant effects in 12 channels (channels: 1: *t* = 3.58, *P* = 0.002; 2: *t* = 3.89, *P* < 0.001; 4: *t* = 3.32, *P* = 0.003; 5: *t* = 5.43, *P* < 0.001; 8: *t* = 3.69, *P* = 0.0015; 9: *t* = 5.26, *P* < 0.001; 10: *t* = 4.31, *P* < 0.001; 13: *t* = 2.23, *P* = 0.036; 14: *t* = 6.17, *P* < 0.001; 15: *t* = 4.32, *P* < 0.001; 28: *t* = 6.51, *P* < 0.001; 32: *t* = 5.36, *P* < 0.001; and 33: *t* = 4.00, *P* < 0.001). Furthermore, for both conditions, activation was evident in the regions of interest (left and right pSTS-TPJ region). The significant HHb effects did not survive the multiple comparison analysis. The main group effects were consistent with previous findings (Lloyd-Fox et al. 2009, 2011), revealing activation to the perception of manual actions and eye gaze shifts in inferior frontal and temporal regions of the cortex. A more widespread pattern of activation was observed in the left hemisphere than the right.

**Figure 3.**
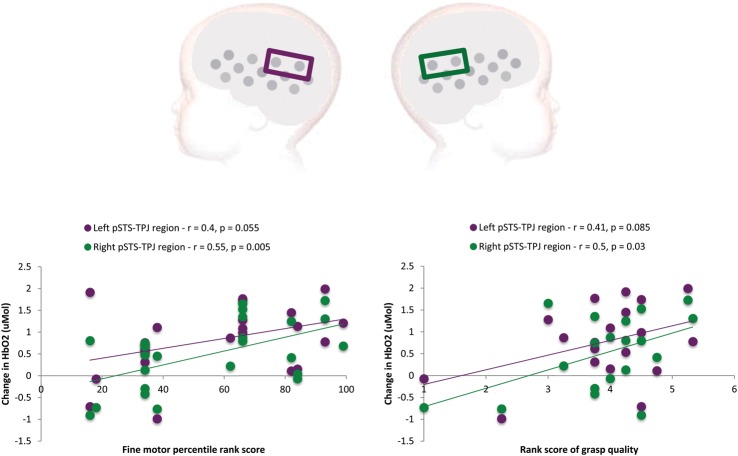
The upper panel illustrates the location of the pSTS-TPJ region of interest in each hemisphere (purple refers to the left hemisphere, and green refers to the right hemisphere). The lower panel illustrates the individual infants' fine motor Mullen scores (left) and score on the Manual Dexterity Task (right) compared with the peak change in HbO_2_ in the left (purple) and right (green) ROIs in response to the perception of the manual action condition.

In a secondary analysis, ROIs for the pSTS-TPJ region were used to compare activation to the conditions with the behavioral measures of fine motor development. These behavioral measures included the Mullen Scales for Early Learning (Mullen 1995) and a Manual Dexterity task (detailed in the Methods section). The peak change in HbO_2_ in each ROI for each individual was compared with his or her fine motor score (see Fig. 3 for plots of the correlations). A Pearson's correlation coefficient analysis revealed a significant correlation between the change in HbO_2_ in the right pSTS-TPJ region in response to the manual action condition and the Mullen fine motor percentile rank score (*r* = 0.55, *P* = 0.005, 2-tailed). Furthermore, the response was also correlated with the quality of grasp (*r* = 0.497, *P* = 0.03, 2-tailed) used during the Manual Dexterity task (with the lowest scoring (grasp quality) infant removed the *P* value is reduced to 0.122). In addition, there was a near significant trend for a correlation between the change in HbO_2_ in the left pSTS-TPJ region in response to the manual action condition and the Mullen fine motor percentile rank score (*r* = 0.40, *P* = 0.055, 2-tailed) and the quality of grasp (*r* = 0.405, *P* = 0.085, 2-tailed) during the Manual Dexterity task. There were no significant correlations in the ROIs for the cortical response to the eye gaze condition and behavioral measures. To examine the specificity of the effect, we investigated similar correlations in 2 other ROIs in each hemisphere; an inferior frontal ROI (LH channels 1 and 4; *r* = 0.09, *P* = 0.69, RH channels 14 and 17; *r* = 0.24, *P* = 0.26) and an anterior temporal ROI (LH channels 5 and 8; *r* = 0.16, *P* = 0.45, RH channels 18 and 21; *r* = 0.2, *P* = 0.36). No significant correlations were found.

To investigate whether age or cognitive ability contributed to these findings, the measures of cortical activation were compared with the overall Mullen standard score of the Mullen Scale of Early Learning (which include visual receptive, fine motor, expressive, and receptive language components). A partial correlation analysis, controlling for age, revealed a significant correlation for the Mullen standard score with the change in HbO_2_ in the right pSTS-TPJ region in response to the manual action condition (*r* = 0.455, *P* = 0.029, 2-tailed). To investigate this effect further, a subsequent exploration of correlations of the other subscales of the Mullen and activation to the manual action condition in the right pSTS-TPJ region revealed no correlation with the visual receptive (*r* = 0.141, *P* = 0.51) or expressive language (*r* = −0.01, *P* = 0.96) percentile rank scores, but there was a significant correlation with the receptive language score (*r* = 0.473, *P* = 0.02). This suggests that the scores from the fine motor and receptive language (which for 4- to 6-month olds largely concerns social interaction, measuring responses to voice and face; reorienting/smiles/giggles, and vocalizations/smiles during self-mirror interaction) scales were driving the overall correlation reported for the Mullen standard score. Note that while a correlation with the receptive language score was also evident for the manual action condition in the left pSTS-TPJ region (*r* = 0.409, *P* = 0.047), there were no significant correlations for the receptive language score and eye gaze condition in either ROI (left ROI: *r* = −2.13, *P* = 0.318; right ROI: *r* = 0.109, *P* = 0.612).

Finally, there was no correlation—in either ROI—for the cortical response to the manual action condition and the gross motor scale of the Mullen (left ROI: *r* = −0.014, *P* = 0.947; right ROI: *r* = 0.039, *P* = 0.858). This suggests that the observed association is not explained by individual variation in general motor development.

To assess infants' attention to the stimulus during the fNIRS session, eye-tracking data were simultaneously collected and later analyzed. Partial correlation analyses were conducted to compare the cortical responses in the pSTS-TPJ ROI and the proportion of time spent looking at the hand/face ROIs, while controlling for total average looking time per condition. For the infants in the group that had good quality eye-tracking data (*N* = 16), there was no correlation between the measures of individual cortical activation in the ROIs and the proportion of time per trial spent looking at the hand during the manual action condition (left ROI: *r* = 0.31, *P* = 0.26; right ROI: *r* = 0.32, *P* = 0.24). However, there was a significant correlation between the proportion of time spent looking at the face and the measures of individual cortical activation during the eye gaze shift condition for one hemisphere (left ROI: *r* = 0.59, *P* = 0.021) but not the other (right ROI: *r* = 0.35, *P* = 0.2). Finally, to assess whether there is a direct link between infant's motor skills and how they attend to the perception of the manual action condition a partial correlation analysis was conducted to compare looking time to the stimulus with fine motor development. There was no correlation between the fine motor scale of the Mullen or the quality of grasp in the Manual dexterity task and the proportion of time spent looking at the hand ROI (fine motor: *r* = 0.11, *P* = 0.68; grasp quality: *r* = 0.05, *P* = 0.87) or the average total looking time (fine motor: *r* = 0.18, *P* = 0.51; grasp quality: *r* = 0.1, *P* = 0.75) during the manual action condition. For further detailed description of the individual behavioral data, see Supplementary Information (Supplementary Table 2).

## Discussion

In this study, we investigated the neural processing resulting from viewing manual actions and the association with developing fine motor skills in human infants. The main group effects in response to the perception of human actions were consistent with previous findings in infants (Lloyd-Fox et al. 2009, 2011) and adults (for a review see Allison et al. 2000; Pelphrey et al. 2005), revealing activation to the perception of viewed manual actions and eye gaze shifts in bilateral inferior frontal and posterior temporal regions of the cortex. Furthermore, as suggested by the preliminary findings in a previous study on action perception (Lloyd-Fox et al. 2011), individual differences in patterns of activation to the manual actions were apparent across the group of infants. The eye-tracking data confirmed that the individual patterns of cortical activation were not driven by the proportion of each infant's looking time to the hand or face during each of the conditions. In other words, the individual differences observed in brain responses across the group of infants were not simply a reflection of their differences in global attention.

When we focused on the analysis of individual differences in activation in the pSTS-TPJ region and behavior, we found that 4-to-6-month-olds' hemodynamic responses to the perception of manual actions were associated with their own developing fine motor skills. Cortical responses in the pSTS-TPJ region, particularly in the right hemisphere, showed a significant correlation with the degree of fine motor skill development. Strikingly, the measures of cortical activation to the perception of manual actions were not correlated with gestational age, or attention to the stimulus, and were only partially associated with developmental age (though this measure, taken from the Mullen standard score, was largely driven by the score in the subscale of fine motor development). Nor were such findings evident for the eye gaze shift condition.

What could account for the co-development of neural responses to perceiving action and the individual's own motor skills? It is difficult to say in this context, whether the very experience of using your hands more—and developing your manual expertise—may provide the richest form of visual self-experience of manual actions. Indeed given that infants' perceptual and motor abilities intertwine while they explore their surroundings, the development of perception and action are difficult to dissociate (Soska et al. 2010). In a study of an adult who is a congenital amputee, researchers showed that activation to the perception of actions was modulated both by the individual's self-experience of actions and by their experience of observing actions which are outside their own motor repertoire (Aziz-Zadeh et al. 2012). It is likely that emerging perceptual skills guide exploration of the environment, while the interpretation of visual cues simultaneously becomes more sophisticated with the acquisition of motor skills. Rather than a cascade of developmental steps, visual and motor self-experience both contribute to the development of our cognitive capacity to interpret the relevance of perceived actions in others.

We found no relationship between general motor development (as measured by the gross motor component of the Mullen Early Learning Scale, which in this age band looks at body posture, neck strength, sitting ability, etc.) and cortical activation in the pSTS-TPJ region to the perception of manual movement in others. While previous behavioral research has shown that expansion of hand use and the mapping of prehensile space is related to self-sitting ability (Rochat and Goubet 1995), the current findings would suggest that a general improvement in body posture or self-sitting ability is not fundamentally responsible for the association of fine motor experience and cortical responses in the pSTS-TPJ region. Perhaps during the first few months of life, while in a supine position or held by an adult, infants' explorations with their own hands—both as an object of interest and to use to reach for objects of interest in their environment—is sufficient to drive the relationship between action perception in the pSTS-TPJ region and manual dexterity. These findings are in line with recent research (Soska et al. 2010) suggesting that visual-manual exploratory skills are a stronger predictor than self-sitting ability for performance on a 3D object completion study in infants from 4 to 7 months of age.

These results support a close relationship between the recruitment of cortical regions in the pSTS-TPJ cortex in response to the perception of manual movements in others, and the development of one's own manual dexterity in early infancy. As the association was not evident during the eye gaze shift condition, it is unlikely to be related to a general visual response to the perception of human movement. It is possible that infants who are experienced with performing manual actions allocate increased attention to others' performing manual actions, which in turn enhances activation in the pSTS-TPJ region. This is in line with recent findings in adults that evidenced modulation of STS activation—to biological motion and action recognition—by a change in attention (Thompson and Parasuraman 2012). Recent work on the perception of familiar and unfamiliar gestures in adults found that while familiar (relative to unfamiliar) gestures activated the mentalizing system, unfamiliar (relative to familiar) gestures activated the posterior regions of the AON, namely, the IPL (Liew et al. 2011). Further, they found that there was increased activation in the IPL when the actor was the same race, relative to a different race, as the participant. These surprising findings suggest that, depending on the context, the degree of familiarity can cause opposing patterns of activation in regions of the AON. In contrast, activation in the STS was not modulated by either context of familiarity. A recent fNIRS study with infants (Grossmann et al. 2013) found that regardless of form (robot or human) an unfamiliar robotic dance action caused increased activation in the right inferior frontal-premotor region. In contrast, in the left anterior temporal region there was increased activation to the pairing of familiar form and motion (i.e., human form-human action and robot form-robot action) relative to unfamiliar pairings (i.e., human form-robot action). These findings in adults and infants could reflect modulation of activation in different regions of the brain according to the context of the action. We speculate that increased activation occurs both in the context of unfamiliar actions (i.e., robotic dance/unfamiliar gestures) where the attentional demand is greater, and in the context of actions that are relevant to oneself (i.e., own race/congruent form and motion), where tuning to actions may arise through an associative learning mechanism (Press 2011). Therefore, the current findings may reflect increased attention or tuning to actions that infants perceive as relevant to themselves because they too are experienced at performing similar manual actions.

As fNIRS does not currently have sufficient spatial resolution, the activation we observed could be attributed either to the pSTS, TPJ, or IPL (though see Van Overwalle and Baetens 2009). One could argue, given the findings of Cross et al. (2009, 2006), that the association with action production suggests that this pSTS-TPJ region activation derives from the IPL, since activation in the STS to the perception of dance moves in adults was not correlated with previous physical training of the same dance moves, while activation in the IPL was (Cross et al. 2009). To understand how the current pSTS-TPJ region activation relates to this, future work that enhances experience by training infants in the production of novel actions (such as the work of Sommerville et al. 2005) would allow one to investigate such effects of familiarity further. Alternatively, research has suggested that the visual analysis of human actions in the STS and/or TPJ is associated with the processing of intentionality and the social relevance of actions (i.e., Allison et al. 2000; Kampe et al. 2003; Lotze et al. 2006; Saxe and Powell 2006; Schilbach et al. 2006; Capek et al. 2008). It is possible that these cognitive processes (which were not investigated in Cross et al. 2006, 2009) were recruited differentially during the observation of the hand actions according to whether the observer was highly skilled at performing intentional and/or socially relevant actions themselves. However, this remains highly speculative given that the manual actions used in the current study were not goal-directed. Given that this is the first evidence of an association between cortical activation in the pSTS-TPJ region to action perception and self-experience of action production in infancy, further work is necessary before drawing any strong conclusions about the precise origin of this activation.

Interestingly, in a post hoc analysis, we found that the Mullen subcomponent measuring receptive language development was also correlated with increased cortical activation to the perception of manual actions (and not to the perception of eye gaze shifts). At the age of 4–6 months, this component largely concerns social responsiveness (i.e., assesses interactions with self in mirror, with others during Peek-a-boo; and responses to familiar words and sounds). Though, we acknowledge that the observed manual actions in the current study were not goal-directed in the classic object-based sense. We speculate that the novel isolated manual movements and accompanying direct gaze may have triggered processing of the social relevance or intentionality of the actions—resulting in an increased cortical response in the pSTS-TPJ region—in those infants who were more “socially responsive.” In contrast, we propose that the eye movements as presented in our experiment provided weaker ostensive signals for communication or joint attention and, therefore, the activation in this condition did not correlate with the infant's own development. It is possible that infants quickly perceived that the repeated gaze movements were not goal oriented or communicatively relevant. Because our study did not vary the degree of social communicative cues or attention getting directly, our speculation about the correlation with the receptive language component remains tentative and should be explored in future work.

How might behavioral findings in goal-directed action studies in infants relate to the current findings? Some have proposed that with the discovery that you can manipulate your limbs to achieve goals (as in the current fine motor tasks which assessed object-directed actions), comes an improved ability to interpret the relevance of others goal-directed actions (Sommerville et al. 2005; Daum et al. 2011) and identify physically impossible actions (Reid et al. 2005). Whether the pSTS-TPJ regional activation observed in the current study would be modulated by the interpretation of goals when watching the goal-directed actions of others has not yet been studied in infants. It is possible that the development of fine motor skills in young infants allows them to see the world as a place where actions are generated by goals, and that this accounts for the differential activation in the pSTS-TPJ region in this study. In other words, an infant's ability to achieve goals via their own actions motivates them to search for goals when watching the actions of others. While we can presently only speculate on such associations, the availability of infant MRIs and age appropriate templates will allow more precise localization of NIRS channels with underlying anatomy. Therefore, future studies will allow us to investigate the role of the pSTS-TPJ region further, and bring together the findings from the current study with previous behavioral research.

Finally, these findings might have important implications for trajectories of atypical development in which both motor and social skills can be affected (Hansen et al. 2010; Hill 2010; Iverson 2010). By adopting a longitudinal design, the current method could be used to assess individual differences in infants' responses, and may contribute to the elucidation of early markers of infants at risk for a neurodevelopmental disorder (Elsabbagh and Johnson 2010) and subsequently to the development of interventions.

## Supplementary Material

Supplementary material can be found at: http://www.cercor.oxfordjournals.org/.

## Funding

This work was supported by the UK Medical Research Council (G0701484) and the National Institutes of Health, National Institute of Child Health and Human Development (#R37 HD19842). Funding to pay the Open Access publication charges for this article was provided by the UK Medical Research Council (G0701484).

## Supplementary Material

Supplementary Data
