# Supplementary material for: Cortical Activation to Action Perception is Associated with Action Production Abilities in Young Infants
Source: Cereb Cortex. 2013 Aug 23;25(2):289–97. doi: 10.1093/cercor/bht207 (PMC4303799; doi:10.1093/cercor/bht207)
Supplement: Supplementary Data [file supp_25_2_289__index.html]

Cortical Activation to Action Perception is Associated with Action Production Abilities in Young Infants — Cortical Activation to Action Perception is Associated with Action Production Abilities in Young Infants — Cortical Activation to Action Perception is Associated with Action Production Abilities in Young Infants — Supplementary Data 

# Cortical Activation to Action Perception is Associated with Action Production Abilities in Young Infants

## Supplementary Data

Supplementary Data

**Files in this Data Supplement:**

- Supplementary Table 1 - pdf file
- Supplementary Table 2 - pdf file
- Supplementary Data - Docx file
- Supplementary Figure 1 - tif file
