# Supplementary material for: Cortical Activation to Action Perception is Associated with Action Production Abilities in Young Infants
Source: Cereb Cortex. 2013 Aug 23;25(2):289–97. doi: 10.1093/cercor/bht207 (PMC4303799; doi:10.1093/cercor/bht207)
Supplement: Supplementary Data [file supp_bht207_bht207supp_table1.pdf]

Manual Action Condition				Eye Gaze Condition			
**Ch**	*t*	*p*	*df*	**Ch**	*t*	*p*	*df*
**1**	3.63	0.001	*23*	**1**	3.58	0.002	*22*
**5**	2.85	0.009	*22*	**2**	3.89	<0.001	*23*
**9**	3.58	0.002	*21*	**4**	3.32	0.003	*23*
**13**	3.34	0.003	*23*	**5**	5.43	<0.001	*22*
**14**	4.81	<0.001	*23*	**8**	3.69	0.0015	*19*
**15**	4.93	<0.001	*23*	**9**	5.26	<0.001	*21*
**28**	6.67	<0.001	*14*	**10**	4.31	<0.001	*22*
**32**	5.14	<0.001	*23*	**13**	2.23	0.036	*23*
				**14**	6.17	<0.001	*23*
				**15**	4.32	<0.001	*23*
				**28**	6.51	<0.001	*14*
				**32**	5.36	<0.001	*23*
				**33**	4.00	<0.001	*23*
