# Supplementary material for: Cortical Activation to Action Perception is Associated with Action Production Abilities in Young Infants
Source: Cereb Cortex. 2013 Aug 23;25(2):289–97. doi: 10.1093/cercor/bht207 (PMC4303799; doi:10.1093/cercor/bht207)
Supplement: Supplementary Data [file supp_bht207_bht207supp_table2.pdf]

	Mullen Scale of Early Learning (percentile rank)		Manual Dexterity Task (average rank score)	Proportion of time look at ROI in manual action condition ^		Proportion of time look at ROI in eye gaze condition ^	Average total look per valid trial per condition *	
Age	*Standard*	*Fine motor*	*Grasp quality*	*Hand*	*Face*	*Face*	*Manual action*	*Eye gaze*
174	89	16	4.5	n/a	n/a	n/a	n/a	n/a
143	113	84	4	n/a	n/a	n/a	n/a	n/a
153	92	16	4.25	n/a	n/a	n/a	n/a	n/a
153	89	34	3.75	0.68	0.374	0.824	9.3	7.75
174	89	34	3.75	0.273	0.784	0.945	7.24	6.48
149	96	18	1	0.153	0.815	0.944	5.19	5.39
138	110	66	3.75	0.541	0.373	0.756	6.78	6.85
156	95	82	4.75	n/a	n/a	n/a	n/a	n/a
141	103	66	3	n/a	n/a	n/a	n/a	n/a
160	98	62	3.25	n/a	n/a	n/a	n/a	n/a
156	102	34	n/a	n/a	n/a	n/a	n/a	n/a
167	95	34	4.25	0.264	0.621	0.829	7.69	6.51
144	107	66	4.5	0.515	0.429	0.837	9.34	6.12
167	117	98	n/a	0.192	0.733	0.934	7.15	6.9
134	103	38	n/a	0.155	0.764	0.965	7.25	7.93
162	98	34	n/a	0.309	0.633	0.887	8.32	6.64
148	113	84	n/a	0.25	0.556	0.792	8.23	8.16
172	116	93	5.33	0.328	0.613	0.97	6.7	6.94
174	115	82	4.25	0.239	0.727	0.952	7.24	6.76
146	111	66	4.5	0.531	0.371	0.786	7.67	6.37
150	107	66	4	0.71	0.29	0.895	9.59	8.06
153	95	34	3.75	0.32	0.446	0.844	5.48	5.57
144	103	38	2.25	0.209	0.559	0.209	7.54	7.97
158	101	93	5.25	n/a	n/a	n/a	n/a	n/a
