# Supplementary material for: Cortical Activation to Action Perception is Associated with Action Production Abilities in Young Infants
Source: Cereb Cortex. 2013 Aug 23;25(2):289–97. doi: 10.1093/cercor/bht207 (PMC4303799; doi:10.1093/cercor/bht207)
Supplement: Supplementary Data [file supp_bht207_bht207supp.docx]

**Supplementary data**

Table 1: The group results from the t-test channel-by-channel analysis for the two experimental conditions. For each contrast the results for the significant increases in HbO_2_ that survive a multiple comparisons analysis (False Discovery Rate threshold for 26 channels) are displayed, Please note that the degrees of freedom may vary across channels as individual infants my have invalid data in some channels (this is especially the case for the larger channel separations – 4.5cm – where the signal is weaker in some individuals).

Figure 1: The group results from the t-test channel-by-channel analysis for the two experimental conditions.

Table 2: Individual infants’ behavioural data. Note: ^ - the data in these columns refer to the average proportion of time spent looking at the hand or face during valid trials for the manual action and eye gaze conditions during the valid trials; * - the data in these columns refer to the average total time spent looking at the screen during the valid trials for the manual action and eye gaze conditions.
