# Supplementary figures and images for: Cortical Activation to Action Perception is Associated with Action Production Abilities in Young Infants
Source: Cereb Cortex. 2013 Aug 23;25(2):289–97. doi: 10.1093/cercor/bht207 (PMC4303799; doi:10.1093/cercor/bht207)

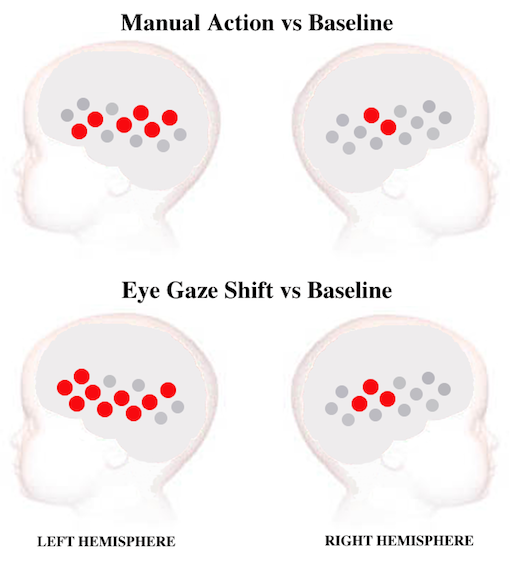

Supplement: Supplementary Data [file supp_bht207_bht207supp_fig1.tif]
